# Right ventricular strain in pulmonary embolism (PE) in Covid-19 pneumonia

**DOI:** 10.5339/qmj.2024.qitc.29

**Published:** 2024-03-26

**Authors:** Muhammad Yousaf, Liaquat Ali, Ahmad A. Abujaber, Salah Almughalles, Saad Rehman, Muhammad Sharif, Syed G Naqvi, Imran Mohammed

**Affiliations:** Hazm Mebaireek Hospital, Hamad Medical Corporation, Doha, Qatar; Weill Cornell Medicine, Cornell University, Doha, Qatar Email: myousaf3@hamad.qa; Hamad General Hospital, Hamad Medical Corporation, Doha, Qatar

**Keywords:** Right ventricular strain, Pulmonary embolism, Covid-19 pneumonia, Qanadli score, CT pulmonary angiogram

## Background

Prompt diagnosis and risk stratification play a crucial role in minimizing the likelihood of adverse clinical outcomes and mortality associated with acute pulmonary embolism (PE). We conducted a post hoc analysis of our published study on PE in Covid-19 pneumonia.^1^ The objective was to assess the incidence of right ventricular (RV) strain and its association with mortality and clot burden, as determined by a Qanadli score.

## Methods and Results

**Figure 1.**
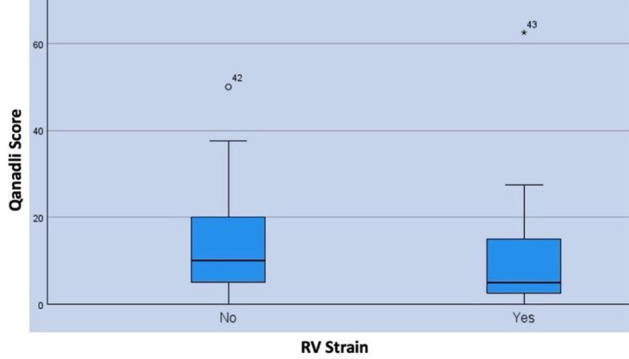
Clot burden (Qanadli score) similarly spread out around the median in each group, regardless of the presence or absence of an RV strain.

The study^1^ had a population of 153 Covid-19 cases with a reported incidence of PE at 41.8%. We analyzed 63 cases of PE in patients with Covid-19 pneumonia. The mean age of the sample was 52.9 ± 10.2 years. According to the American Thoracic Society guidelines for pneumonia, 83% of cases were categorized as critical pneumonia. We defined RV strain on CT pulmonary angiogram (CTPA) by an increased RV/LV ratio of ≥ 1.0, evaluated by two independent radiologists. The analysis revealed an RV strain incidence of 22.2% and a mortality rate of 25.4%. Both logistic regression and univariate chi-square test did not show any significant association between RV strain and Qanadli score (Figure 1). Additionally, there was no association between RV strain and mortality or age. Another study reported similar results,^2^ while others reported variable outcomes. A review of PE distribution and its relationship to RV strain revealed that peripheral PE does not entail a lower risk of RV strain than central PE. The incidence of central PE in our cohort was 11%.

## Conclusion

We found no statistically significant association between RV strain and clot burden in PE in Covid-19 pneumonia. Recognizing the need for a larger and more representative sample is imperative to comprehensively address the pertinent question raised by this study.

## Conflict of Interest

The authors have no conflicts of interest to declare.
